# Coherent control of optical polarization effects in metamaterials

**DOI:** 10.1038/srep08977

**Published:** 2015-03-10

**Authors:** Seyedmohammad A. Mousavi, Eric Plum, Jinhui Shi, Nikolay I. Zheludev

**Affiliations:** 1 Optoelectronics Research Centre and Centre for Photonic Metamaterials, University of Southampton, SO17 1BJ, UK; 2 Key Laboratory of In-Fiber Integrated Optics of Ministry of Education, College of Science, Harbin Engineering University, Harbin 150001, China; 3 Centre for Disruptive Photonic Technologies, Nanyang Technological University, Singapore 637378, Singapore

## Abstract

Processing of photonic information usually relies on electronics. Aiming to avoid the conversion between photonic and electronic signals, modulation of light with light based on optical nonlinearity has become a major research field and coherent optical effects on the nanoscale are emerging as new means of handling and distributing signals. Here we demonstrate that in slabs of linear material of sub-wavelength thickness optical manifestations of birefringence and optical activity (linear and circular birefringence and dichroism) can be controlled by a wave coherent with the wave probing the polarization effect. We demonstrate this in proof-of-principle experiments for chiral and anisotropic microwave metamaterials, where we show that the large parameter space of polarization characteristics may be accessed at will by coherent control. Such control can be exerted at arbitrarily low intensities, thus arguably allowing for fast handling of electromagnetic signals without facing thermal management and energy challenges.

Conventional electromagnetic materials are often used in bulk form, while many useful functionalities can be achieved with very thin layers of metamaterials, artificial media structured on the sub-wavelength scale. For example, ultra-thin absorbers^1^, spectral filters^2^,^3^, wave plates^4^, linear polarizers^5^, highly birefringent hyperbolic materials^6^, optically active materials^7^,^8^,^9^,^10^,^11^,^12^, phase gradient surfaces^13^ and lenses^14^,^15^ have been demonstrated. This provides an opportunity to exploit coherent optical effects, which are now attracting considerable attention as new means of controlling the interaction of electromagnetic fields with photonic structures^16^,^17^,^18^,^19^,^20^,^21^,^22^,^23^,^24^,^25^,^26^, including control of intensity^27^ and direction^28^ of electromagnetic waves at arbitrarily low intensities down to the quantum regime^29^.

We demonstrate control of polarization effects in thin layers of material by engaging interference of two coherent waves on the slab. Recently, we reported first evidence of coherent polarization control in anisotropic and intrinsically 3D-chiral metamaterials^30^. Here we present a more comprehensive analysis which includes intensity measurements, considers oblique incidence, relates both output beams, and extends to coherent polarization control in extrinsically 3D-chiral systems for the first time.

Fig. 1 illustrates the interference of two coherent waves of equal intensity. A thin slab of material placed at an electric anti-node will experience twice the electric excitation field compared to single beam illumination, while no electric excitation can occur if the slab is placed at an electric-field node. Similarly, functional materials placed at magnetic-field anti-nodes or nodes will experience enhanced or zero magnetic excitation, respectively. As electric nodes correspond to magnetic anti-nodes and vice versa, this provides a spectroscopic opportunity to excite thin materials either electrically or magnetically^31^ and it allows enhancement and complete cancelation of any optical phenomena that are controlled by either electric or magnetic excitation. Here we explore how interaction of two waves on a thin sheet of material can be used to control the polarization effects associated with birefringence and optical activity of the slab.

Linear birefringence and linear dichroism are the optical manifestations of anisotropy and lead to phase delays and differential transmission of the ordinary and extraordinary linear eigenpolarizations of anisotropic materials, see Fig. 2a. Optical anisotropy is found in many crystals (e.g. calcite, quartz and magnesium fluoride) and linear birefringence is exploited in wave plates^4^, while linear dichroism is the basis of thin film linear polarizers^5^.

Optical activity manifests itself as circular birefringence and circular dichroism, which rotate the polarization azimuth of electromagnetic waves and change their ellipticity. Conventionally, optical activity is seen in intrinsically 3D-chiral materials such as solutions of twisted organic molecules (e.g. proteins, DNA, sugar molecules), chiral crystals (e.g. quartz, tartaric acid) and artificial helical structures^33^,^34^,^35^,^36^, see Fig. 2b. Particularly large optical activity in thin structures has been observed for intrinsically 3D-chiral stereometamaterials based on pairs of identical, mutually twisted metal patterns in parallel planes^7^,^8^,^37^,^38^,^39^,^40^. It is less well-known that circular birefringence and dichroism can also occur in achiral materials, if chirality resides in the mutual arrangement of wave and material structure (screw directions)^41^,^42^. Manifestations of such “extrinsic” chirality can be easily observed in metamaterials^11^,^12^, and occur for oblique incidence onto planar patterns lacking twofold rotational symmetry, see Fig. 2c.

The coherent interaction of electromagnetic waves on thin functional materials can be described by Jones transmission and scattering matrices, as presented in the Supplementary Information.

## Results

We study coherent polarization control for three well-understood metamaterials that are representative of thin anisotropic materials and thin materials exhibiting optical activity due to intrinsic and extrinsic 3D chirality (Figs 3–6, Refs. 11, 12, 38). All structures are non-diffracting and at least 15× thinner than the wavelength throughout our experiments, see Methods for details. For each case, we first confirm the structure's known linearly birefringent or optically active properties in single beam transmission experiments, and then add a second coherent illuminating beam of equal polarization and intensity to investigate coherent control of the metamaterial's functionality. All intensities are given relative to the intensity of one illuminating beam (100%), so that the total input intensity in experiments with two incident beams corresponds to 200%.

### Coherent control of manifestations of linear birefringence

Linear birefringence and dichroism are studied for an anisotropic array of wire split ring resonators, see Fig. 3a. For achiral experimental arrangements such as normal incidence, the structure has ordinary and extraordinary eigenpolarizations oriented parallel and perpendicular to the pattern's line of mirror symmetry. As for linearly birefringent crystals, the ordinary and extraordinary polarizations will not be changed by the metamaterial, but the polarization effects associated with optical anisotropy can be easily studied for illumination with an intermediate polarization state. Therefore we study optical anisotropy for the wire split ring metamaterial by illuminating it with waves polarized at 45° to its line of mirror symmetry under quasi-normal incidence conditions (an achiral configuration^11^ where the pattern's line of symmetry coincides with the plane of incidence with angle of incidence *θ* = 13°). This is just like one would illuminate a linearly birefringent quarter wave plate to create circular polarization. As illustrated by Fig. 3b, under these illumination conditions, the anisotropic metamaterial dramatically changes the azimuth and ellipticity angle of the transmitted beam across almost the entire investigated spectral range. In particular, it behaves like a quarter wave plate around 9 GHz, where the transmitted beam has left-handed circular polarization.

Here we study how the anisotropic metamaterial response is affected by an additional coherent control beam of the same polarization as the signal beam, see Fig. 3c. Intensity and polarization of the detected signal output beam strongly depend on the phase difference *α* between the control and signal input beams. For in-phase electrical excitation (*α* = 0°) of the metamaterial by the signal and control beams the electric excitation field doubles, leading to a corresponding increase of the structure's scattered fields. This is clearly seen in linear polarization conversion, which originates from the scattering properties of the birefringent metamaterial. In comparison to single beam excitation, the measured conversion to the perpendicular linear polarization approximately doubles in terms of fields, corresponding to an approximately fourfold intensity increase. On the other hand, for anti-phase excitation (*α* = 180°), no polarization conversion has been detected and the signal output intensity is about 100%. Here, destructive interference of the incident electric fields prevents excitation of the metamaterial structure, effectively rendering it transparent at all frequencies. The proportionality of metamaterial excitation and scattering to the electric excitation field *E*_||_, which is controlled by interference of the input beams, is reflected by the sinusoidal phase dependence of the output intensities. In case of polarization conversion this leads to an output intensity proportional to 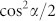. The anisotropic polarization conversion and the associated polarization azimuth rotation and ellipticity changes are non-resonant and therefore broadband and low loss, despite the presence of the lossy dielectric substrate. As a result, the metamaterial acts as a broadband coherent polarization rotator that uniquely maps phase onto polarization azimuth from 6.5 GHz to 11.5 GHz. This is illustrated in detail by Fig. 3d for 9.1 GHz where the ellipticity of the output polarization remains small. Similarly, the structure acts as a coherent ellipticity modulator between 6 and 7 GHz, which allows the output beam to be continuously tuned from right-handed circular polarization to left-handed almost circular polarization.

### Coherent control of manifestations of optical activity due to intrinsic 3D chirality

Optical activity due to intrinsic 3D chirality was studied for a metamaterial based on mutually twisted metal patterns in parallel planes at close to normal incidence (*θ* = 0° without control beam, *θ* = 13° with control beam), see Fig. 4. In contrast to the previous case, the metamaterial's optical properties are polarization azimuth independent at normal incidence due to its fourfold rotational symmetry and only weakly depend on the angle of incidence for small incidence angles. As shown by Fig. 4b, the metamaterial exhibits large circular birefringence and circular dichroism near its resonances at about 4.9 and 6 GHz and *y*-to-*x* polarization conversion peaks at the 4.9 GHz resonance reaching about 12% of the incident intensity. The structure's properties are consistent with those described in Refs 7 and 38, where a negative index of refraction due to giant optical activity has been reported for similar metamaterial designs.

Here we study how the optically active metamaterial response is affected by an additional coherent control beam of the same polarization as the signal beam, see Fig. 4c. The results shown here correspond to s-polarization, however, the metamaterial's optical properties for p-polarization were found to be almost identical. Similarly to the previous case, the polarization state of the signal output beam strongly depends on the phase difference between the input beams. However, in contrast to the planar metamaterial discussed above, there is no special phase *α* where all polarization effects vanish. At the 4.9 GHz resonance, our measurements and simulations show that *y*-to-*x* polarization conversion is non-zero at all phases and it peaks in our experiments near the magnetic anti-node, where electric excitation of the metamaterial vanishes (*α* = 180°). This suggests that magnetic coupling to the metamaterial plays an important role, and indeed, the 4.9 GHz resonance has previously been identified as a magnetic resonance leading to a negative effective permeability and negative refractive index^7^,^38^. The associated mode is characterized by counterpropagating currents in the metal patterns on opposite sides of the substrate (inset to Fig. 4b). Such a magnetic resonance can be excited effectively by the magnetic anti-node, where the magnetic field oscillates with maximum amplitude within the metamaterial plane. Notably, coherent control of the metamaterial's electric and magnetic responses inevitably translates into coherent control of any associated phenomena such as refraction, implying coherent control of negative refraction at the 4.9 GHz negative index resonance. At this resonance, the metamaterial also acts as a coherent polarization rotator that uniquely maps the phase *α* onto the polarization azimuth of the (strongly elliptically polarized) output beam, see Fig. 4d.

We note that simulations and experiments show the same qualitative behavior. The quantitative differences in Fig. 4d may be due to the slightly different angles of incidence in coherent control simulations (0°) and experiments (13°), resulting from having to place the control and receiving antennas side by side.

### Coherent control of manifestations of optical activity due to extrinsic 3D chirality

**Figure 1:**
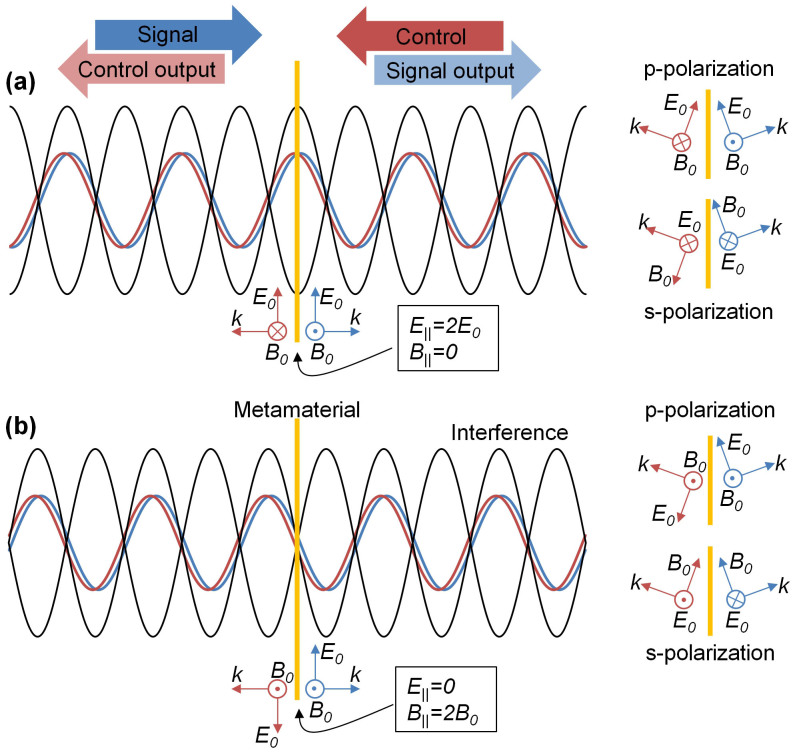
Coherent control of metamaterial functionalities. Coherent counterpropagating beams “Signal” and “Control” form an interference pattern. A functional material of substantially subwavelength thickness can be placed at a position of (a) constructive interference or (b) destructive interference of the incident electric fields *E*_0_ leading to enhanced or vanishing electric excitation of the material, respectively. Similarly, (a) destructive and (b) constructive interference of the incident magnetic fields *B*_0_ leads to vanishing or enhanced magnetic excitation of the metamaterial. Insets illustrate interference for p and s-polarization at oblique incidence.

With respect to extrinsic chirality, it is important to note that the coherent control concept can also be applied at oblique incidence as the constant phase difference *α* between both incident beams at the metamaterial plane is preserved for coherent beams incident in the same plane at the same angle *θ* on the same side of the metamaterial's surface normal. In this case, the electric and magnetic field components parallel to the metamaterial plane interfere in the same way as at normal incidence (*E*_||_, *B*_||_), and new field components normal to the metamaterial plane appear (*E*_⊥_, *B*_⊥_) depending on the incident polarization, compare Fig. 1 and Table 1.

**Figure 2:**
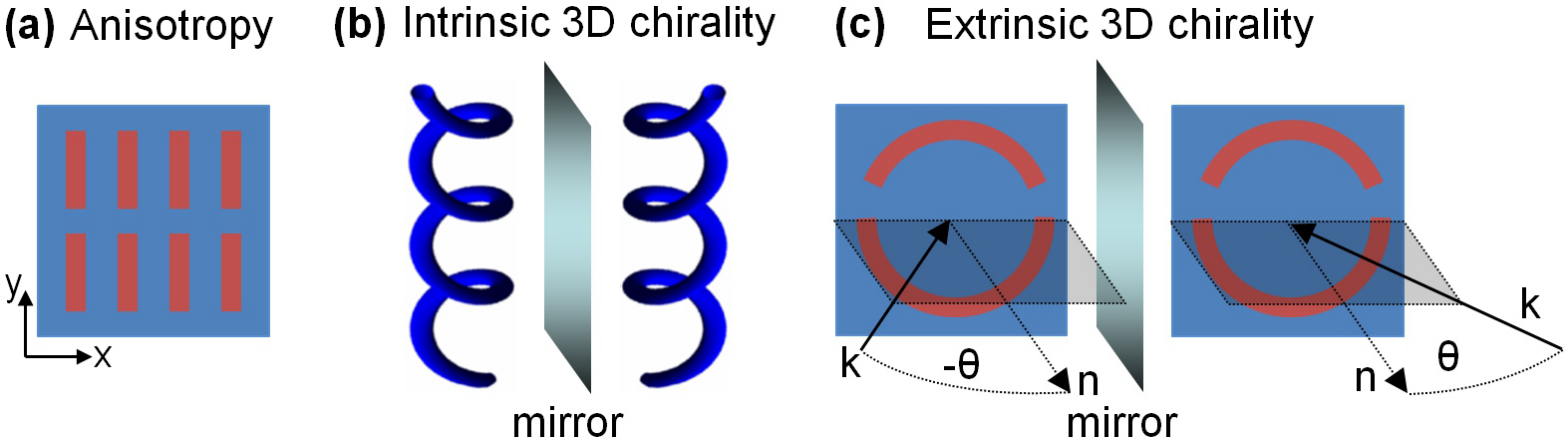
Anisotropy and chirality. (a) Materials that have preferred directions are anisotropic and can show linear birefringence and dichroism. (b) Molecules that cannot be superimposed with their mirror image have intrinsic 3D chirality and their solution can show optical activity^32^. (c) 3D chirality is also present and can also lead to circular birefringence and dichroism when the experimental arrangement consisting of material and direction of incidence *k* cannot be superimposed with its mirror image^11^,^12^.

Metamaterials based on asymmetrically split wire rings and the complementary asymmetrically split ring apertures were studied at oblique incidence where the pattern's line of mirror symmetry was kept perpendicular to the plane of incidence to introduce extrinsic 3D chirality as illustrated by Fig. 2c. By choosing an incident polarization parallel or perpendicular to the metamaterial's line of mirror symmetry we avoid polarization effects linked to the linear anisotropy of the split ring pattern. As shown for the split ring aperture array (s-polarization, Fig. 5b) and the split ring wire array (p-polarization, Fig. 6b) for an angle of incidence of *θ* = 30°, these metamaterials exhibit large optical activity at their respective resonances. Substantial conversion from the incident linear polarization to the perpendicular polarization of about 10% of the incident intensity is seen in both cases and the transmitted wave becomes strongly elliptically polarized with azimuth rotation reaching more than 60°. No polarization conversion or optical activity was seen at normal incidence and opposite circular birefringence and circular dichroism were observed at opposite angles of incidence ±*θ*. The observed optical activity was generally consistent with that reported in Refs. 11, 12 for similar structures.

Here we study how this optically active metamaterial response is affected by an additional coherent control beam of the same polarization as the incident signal beam, see Figs 5c and 6c. Similarly to the achiral configuration discussed above in terms of linear birefringence, both polarization components of the signal output intensity have a sinusoidal phase dependence and the metamaterials are essentially transparent with 100% signal output and complete absence of polarization changes at the electric node (*α* = 180°). Also, polarization conversion in terms of intensity is proportional to 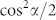, peaking for in-phase electrical excitation (*α* = 0°) with approximately fourfold increased values compared to absence of the control beam. Between the extremes of enhanced and zero coupling to the metamaterials, the relative phase of signal and control beams allows the output azimuth and ellipticity to be controlled over a wide range around the optically active resonances at 9 GHz (Fig. 5c) and 6 GHz (Fig. 6c). In particular, at 9.27 GHz the split ring aperture array acts as a coherent azimuth modulator, which uniquely and approximately linearly maps the phase of the control beam onto the azimuth of the output beam covering the full 180° azimuth range, while the ellipticity angle of the output beam remains relatively small, see Fig. 5d. At 5.88 GHz, the wire split ring metamaterial acts as a coherent ellipticity controller, which controls the ellipticity angle of the output beam continuously from right-handed circular polarization to left-handed circular polarization, see Fig. 6d.

### Control output vs signal output

While only the signal output beam can be measured in our experiments, it is clear that there is a second output beam in the direction of the transmitted control beam, see Fig. 1.

### Planar metamaterials

**Figure 3:**
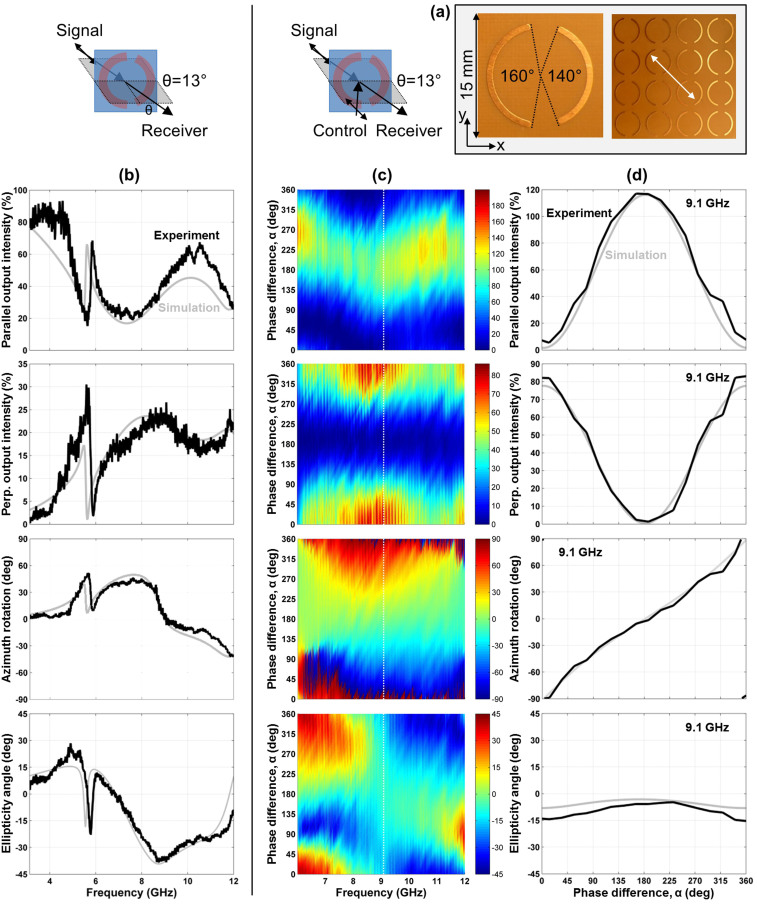
A coherent polarization rotator based on anisotropy. (a) Unit cell and fragment of the metamaterial array consisting of asymmetrically split wire rings with the incident polarization marked by a white double arrow. (b) Transmission characteristics of the metamaterial for 13° oblique incidence of a diagonally polarized signal beam in terms of transmitted intensities polarized parallel and perpendicular to the incident polarization, azimuth rotation and ellipticity angle. (c) Coherent control of these optical properties with an additional control beam polarized parallel to the signal beam as a function of the phase difference *α* between the control and signal beams. (d) The same optical properties for a selected frequency of 9.1 GHz, where the metamaterial behaves as a coherent polarization rotator which can rotate the polarization azimuth continuously over the full 180° range while the ellipticity angle remains small (within ±15°). Solid black lines and color maps show experimental data, while solid gray lines correspond to simulations. The azimuth rotation and ellipticity data shown in the last two rows have been reprinted with permission from Ref. 30. Copyright 2014, AIP Publishing LLC.

As illustrated by Fig. 7a for *planar metamaterials* in general, in comparison to a measurement of the signal output beam, a measurement of the control output beam corresponds to the mirror image experiment with reversed roles of signal and control beams. This implies that the control output at phase *α* corresponds to the signal output at phase −*α* (equivalent to 360° − *α*) with reversed signs of polarization azimuth rotation ΔΦ^c,out^(*α*) = −ΔΦ^s,out^(−*α*) and ellipticity *η*^c,out^(*α*) = −*η*^s,out^(−*α*), as measured looking into the respective beam. This symmetry strictly applies to truly planar metamaterials such as the split ring aperture array, which have reflection symmetry with respect to the metamaterial plane. It becomes an approximation in case of the split ring wire array, where the substrate breaks reflection symmetry with respect to the metamaterial plane. It follows that the anisotropic and extrinsically 3D-chiral planar structures act as coherent polarization rotators and ellipticity controllers simultaneously for both output beams, see Figs 3, 5 and 6.

### Intrinsic 3D chirality

In contrast to planar metamaterials, the signal output and control output beams are more simply related in case of intrinsically 3D-chiral metamaterials, as the 3D-chiral twist is the same for observation from opposite sides. Starting with a measurement of the signal output and assuming polarization azimuth-independent optical properties that are identical for opposite directions of illumination, at normal incidence, a measurement of the control output is equivalent to simply interchanging the roles of signal and control beams, as illustrated for the simplest case of pairs of mutually twisted crosses^39^,^40^ in Fig. 7b. This implies that the control output at phase *α* corresponds to the signal output at phase −*α* with the same signs of polarization azimuth rotation ΔΦ^c,out^(*α*) = ΔΦ^s,out^(−*α*) and ellipticity *η*^c,out^(*α*) = *η*^s,out^(−*α*).

Our experimental 2D-chiral crosses of fourfold rotational symmetry exhibit polarization azimuth-independent optical properties and complete absence of 2D-chiral optical effects at normal incidence^38^,^43^. However, slightly oblique incidence and slightly different resonant reflectivity for opposite directions of illumination resulting from the different orientation of the metal patterns on opposite sides of the dielectric substrate make the above symmetry an approximation near resonances in our experimental case.

### Coherent absorption and coherent intensity modulators

**Figure 4:**
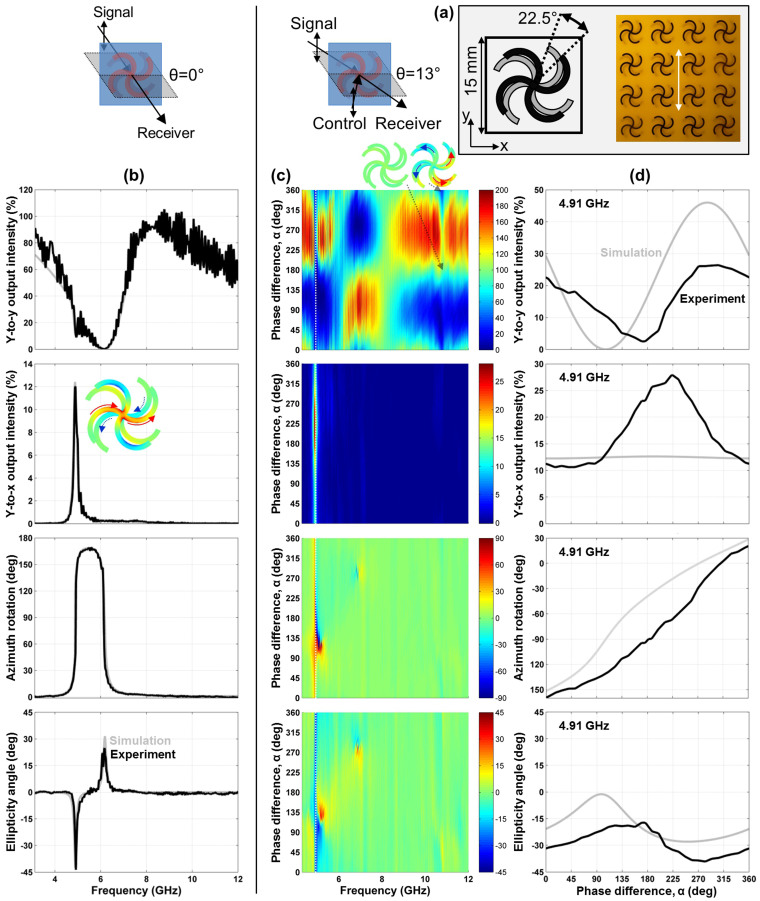
A coherent polarization rotator based on intrinsic 3D chirality. (a) Unit cell and fragment of the metamaterial array consisting of pairs of mutually twisted metal patterns spaced by a dielectric substrate of 1.6 mm thickness with the incident polarization marked by a white double arrow. (b) Transmission characteristics of the metamaterial at normal incidence of a *y*-polarized signal beam in terms of *y*-to-*y* intensity transmission, *y*-to-*x* intensity conversion, azimuth rotation and ellipticity angle of the detected beam. The inset shows the *x*-component of the current density at the 4.9 GHz resonance. (c) Coherent control of these optical properties at close to normal incidence (*θ* = 13°) with an additional *y*-polarized control beam as a function of the phase difference *α* between the control and signal beams. Insets show the *x*-component of the current density at the 10.7 GHz resonance for phases *α* = 0° and 180°. (d) The same optical properties for a selected frequency of 4.91 GHz, where the metamaterial behaves as a coherent polarization rotator which can rotate the polarization azimuth continuously over the full 180°. Solid black lines and color maps show experimental data, while solid gray lines correspond to simulations. The azimuth rotation and ellipticity data shown in the last two rows have been reprinted with permission from Ref. 30. Copyright 2014, AIP Publishing LLC.

**Figure 5:**
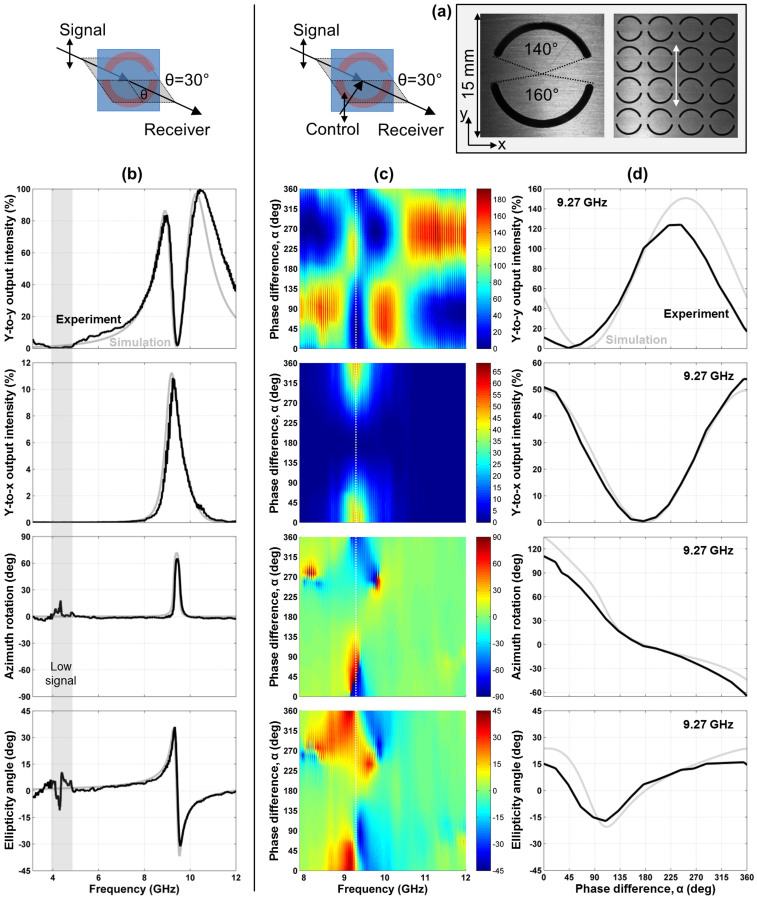
A coherent polarization rotator based on extrinsic 3D chirality. (a) Unit cell and fragment of the metamaterial array consisting of asymmetrically split ring apertures in an aluminum sheet with the incident polarization marked by a white double arrow. (b) Transmission characteristics of the metamaterial for 30° oblique incidence of a *y*-polarized signal beam in terms of *y*-to-*y* intensity transmission, *y*-to-*x* intensity conversion, azimuth rotation and ellipticity angle of the detected beam. (c) Coherent control of these optical properties with an additional *y*-polarized control beam as a function of the phase difference *α* between the control and signal beams. (d) The same optical properties for a selected frequency of 9.27 GHz, where the metamaterial behaves as a coherent polarization rotator which can rotate the polarization azimuth continuously over the full 180° range while the ellipticity angle remains small (within ±15°). Solid black lines and color maps show experimental data, while solid gray lines correspond to simulations.

**Figure 6:**
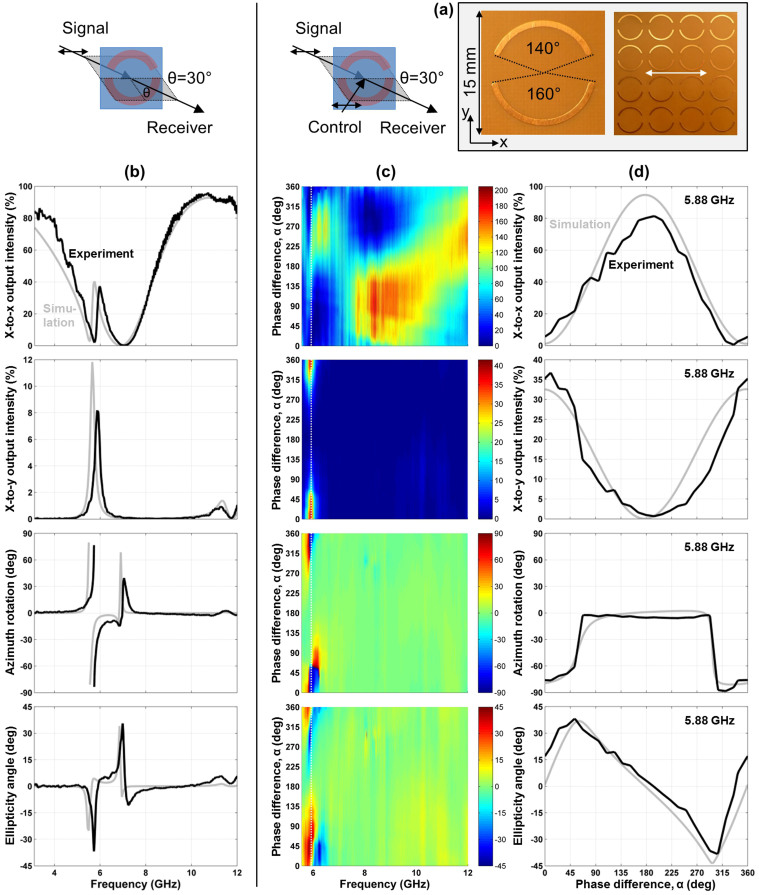
A coherent ellipticity controller based on extrinsic 3D chirality. (a) Unit cell and fragment of the metamaterial array consisting of asymmetrically split wire rings with the incident polarization marked by a white double arrow. (b) Transmission characteristics of the metamaterial for 30° oblique incidence of an *x*-polarized signal beam in terms of *x*-to-*x* intensity transmission, *x*-to-*y* intensity conversion, azimuth rotation and ellipticity angle of the detected beam. (c) Coherent control of these optical properties with an additional *x*-polarized control beam as a function of the phase difference *α* between the control and signal beams. (d) The same optical properties for a selected frequency of 5.88 GHz, where the metamaterial behaves as a coherent ellipticity controller. Solid black lines and color maps show experimental data, while solid gray lines correspond to simulations.

For all considered cases, the control output intensity at phase *α* corresponds to the signal output intensity at phase 360° − *α* and thus the total output power may be calculated by adding up the measured output intensities at these phases. As expected from energy conservation, within experimental uncertainty the intensities of both output beams add up to the combined intensity of both input beams of 200% for the split ring aperture array, which is essentially lossless at microwave frequencies, see Fig. 5c. The same holds for the lossy metamaterials at non-resonant frequencies, see Figs 3c, 4c and 6c.

On the other hand, the wire split ring metamaterial exhibits substantial losses around its resonant region between 5.5 and 7 GHz, where in-phase excitation (*α* = 0°) leads to substantial coherent absorption^27^ in the lossy dielectric substrate, see Fig. 6c. In case of the 4.9 GHz resonance of the intrinsically 3D-chiral metamaterial absorption is large for all values of *α*, indicating that both electric and magnetic excitation fields, *E*_||_ and *B*_||_, strongly couple to the metamaterial, see Fig. 4c.

It is also interesting how the incident power is split between the output beams. For *α* = 180°, each output beam carries about 100% intensity and all polarization effects are absent for all investigated structures, except at resonances of the intrinsically 3D-chiral metamaterial.

Furthermore, all metamaterials can act as coherent intensity modulators, which map the relative phase of the signal and control beams onto the intensity of the output beams. For phases *α* = 90° and *α* = 270°, the intrinsically 3D-chiral metamaterial (7 GHz and 10 GHz), the split ring aperture array (10 GHz and 10.5–12 GHz) and the wire split ring metamaterial (Fig. 6c, 8.4 GHz) direct almost the entire intensity of both input beams into the same output beam without polarization change. Considering a phase modulated signal beam and a reference control beam, this will yield two separate amplified intensity modulated output beams corresponding to the leading and trailing signal phase components, respectively.

## Discussion

For the planar metamaterials, transparency and complete absence of all polarization effects at the magnetic anti-node (*α* = 180°) in all experimental configurations (for both s and p polarizations at normal and oblique incidence for both samples) indicates that tangential magnetic *B*_||_ and normal electric *E*_⊥_ fields cannot excite the planar structures, which should be expected where the motion of electrical charges is confined to a single plane (see Table 1). On the other hand, all planar structures were strongly excited by tangential electric fields *E*_||_ leading to broadband polarization effects resulting from optical anisotropy for achiral quasi-normal incidence illumination with a polarization state containing both ordinary and extraordinary polarization components as well as large resonant optical activity in extrinsically 3D-chiral experimental configurations (where the excitation field was orientated parallel or perpendicular to the pattern's line of symmetry to avoid optical anisotropy effects).

In contrast to the planar structures, resonant optical activity at quasi-normal incidence was observed for a layered intrinsically 3D-chiral structure both at the electric and magnetic anti-nodes, indicating that the optically active resonance at 4.9 GHz can be excited by both tangential electric *E*_||_ and magnetic *B*_||_ fields. Measurements for s and p polarizations at small angles of incidence did yield almost identical results, suggesting that the associated smaller magnetic and electric excitation normal to the metamaterial plane does not strongly affect the metamaterial response. (Except for the absorption resonance without significant polarization changes at 10.7 GHz for *α* = 0° [see insets of Fig. 4c], which is only seen for s-polarization, indicating that it originates from *B*_⊥_ excitation [compare Table 1].)

**Figure 7:**
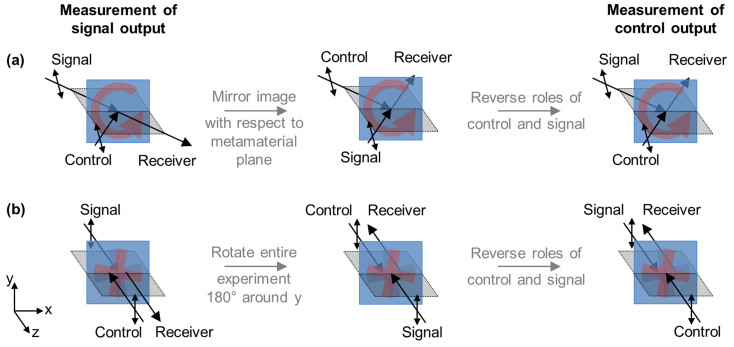
Signal output vs control output. (a) Planar metamaterials: Starting from the signal output for phase *α*, for an arbitrary planar metamaterial and an arbitrary choice of polarization, the control output corresponds to the mirror image experiment (*reversed* azimuth rotation and ellipticity) with reversed roles of signal and control beams (phase −*α*). (b) The simplest case for intrinsic 3D chirality: Starting from the signal output for phase *α*, for normally incident *y*-polarization onto a pair of mutually twisted crosses in parallel planes, the control output corresponds to the same experiment (*same* azimuth rotation and ellipticity) with reversed roles of signal and control beams (phase −*α*). This relationship can be generalized to any choice of linear polarization for metamaterials with polarization azimuth-independent optical properties (e.g. 3-fold or higher rotational symmetry) that are identical for opposite directions of illumination. (a, b) The metamaterial is represented by a unit cell, the plane of incidence is shown by a gray sheet and the polarization state is indicated by a double arrow. The control beam polarization is chosen so that it has the same projection onto the metamaterial plane as the signal beam polarization.

The different relationship between signal output and control output for the intrinsically 3D-chiral metamaterial and the planar metamaterials can be easily understood as follows (compare Fig. 7). For all structures, a measurement of the control output resembles a measurement of the signal output for interchanged input beams, i.e. for the opposite phase difference between signal and control beams. However, while intrinsic 3D chirality is the same for both input beams, for extrinsically 3D-chiral configurations the handedness associated with the directions of signal and control beams is opposite. Similarly, in case of optical anisotropy, where polarization effects occur for incident polarizations that are neither parallel nor perpendicular to the planar pattern's line of mirror symmetry, an excitation polarization azimuth to the right of the pattern's mirror line as seen looking into the signal beam, corresponds to an azimuth to the left of the mirror line when looking into the control beam. Therefore, the control output corresponds to the signal output for the opposite phase with *same* azimuth rotation and ellipticity for intrinsic 3D chirality and *opposite* azimuth rotation and ellipticity for planar metamaterials (extrinsic 3D chirality and anisotropy). These relationships have been confirmed by numerical simulations and theoretical proofs for the chiral cases are given in the Supplementary Information.

While the experimental results reported here were measured in the microwave part of the spectrum, the same concepts can be applied across the electromagnetic spectrum. Optical activity due to intrinsic and extrinsic 3D chirality has also been observed in THz^44^ and optical metamaterials^8^,^9^,^10^,^12^,^40^,^45^ of sub-wavelength thickness and numerous thin anisotropic metamaterial patterns have been reported in the literature^4^,^5^, including a photonic metamaterial for which coherent control of absorption has been reported recently^27^. Coherent excitation of these structures in their respective spectral ranges of operation should lead to similar optical phenomena to those reported here. Modulation based on coherent control may generally be realized by splitting the electromagnetic signal and recombining the separate optical paths on the metamaterial, where the relative phase of control and signal beams can be controlled by translation of the metamaterial with sub-wavelength resolution or by modulating the phase of one beam using a delay line, an electronic phase modulator (for microwaves) or a liquid crystal phase modulator (for the infrared and visible).

Coherent control of polarization states and intensity has tremendous potential for modulation of electromagnetic waves. For non-resonant polarization control (as reported here for optical anisotropy) and non-resonant intensity modulation (as seen in most of our experiments), achievable modulation rates should approach the frequency of the electromagnetic wave itself, while achievable modulation rates for resonant polarization control (as reported here for optical activity) should be lower by about the resonance quality factor. Applied to the optical telecommunications band around 1.5 *µ*m wavelength, where metamaterial resonances typically have quality factors on the order of 10, this promises modulation rates on the order of 10 THz for resonant effects.

More generally, by allowing the selective excitation of materials with electric or magnetic fields tangential to their surface, coherent control provides a useful tool for probing thin samples at normal or oblique incidence. Also selective probing with electric and magnetic fields perpendicular to the surface is possible with incident p and s-polarizations, however, it comes at the cost that only one field component vanishes parallel to the interface, compare Table 1.

## Conclusions

In summary, we show that coherent control of functional materials of subwavelength thickness allows manipulation of the expression of their polarization properties. Coherent control may be achieved by placing the functional material into the standing wave of two counterpropagating coherent beams, where the excitation in the plane of the functional material can be shifted continuously from electric in the anti-node of the electric field to magnetic in the magnetic anti-node by controlling the relative phase of the illuminating beams.

Specifically, we demonstrate coherent control of manifestations of optical anisotropy and optical activity. We report coherent control of polarization effects resulting from optical anisotropy of a planar metamaterial and optical activity of thin intrinsically/extrinsically 3D-chiral metamaterials in the microwave part of the spectrum, demonstrating several coherent polarization rotators and coherent ellipticity controllers. We also demonstrate coherent intensity modulators that can direct almost all input intensity into a single output beam. All observed phenomena can be scaled throughout the electromagnetic spectrum up to the optical spectral range. In particular, non-resonant coherent polarization modulators based on optical anisotropy are promising for practical applications due to their low absorption and large bandwidth. Scaled to optical frequencies, such structures promise energy-efficient polarization control with 10s of THz bandwidth for coherent optical data processing networks.

Furthermore, coherent illumination provides means of selectively exciting thin materials with electric or magnetic fields tangential or perpendicular to their surface, opening up a new opportunity for spectroscopy.

## Methods

### Sample description

We study coherent control of manifestations of optical anisotropy and optical activity for three metamaterials in the microwave part of the spectrum between 3 and 12 GHz (100-25 mm wavelength). All structures have a diameter of 220 mm, a thickness of no more than 1.6 mm and they consist of a square array of 15 × 15 mm^2^ meta-molecules, which renders them thin compared to the wavelength and non-diffracting throughout our experiments. The metamaterials are an array of asymmetrically split wire rings etched on a dielectric substrate (Figs 3 and 6, ring radius 6 mm, line width 0.8 mm, substrate thickness 1.6 mm), the complementary array of ring apertures cut into an aluminum sheet (Fig. 5, ring radius 6 mm, cut width 1 mm, sheet thickness 1 mm) and an intrinsically 3D-chiral array consisting of mutually twisted pairs of identical metal patterns etched on both sides of a dielectric substrate of 1.6 mm thickness (Fig. 4). The latter pattern of fourfold rotational symmetry consists of 4 semicircles of 2.9 mm radius and 0.8 mm line width. Other relevant structural details are marked in the respective figures.

### Experimental characterization

All samples were studied in a microwave anechoic chamber using 3 broadband microwave antennas and a vector network analyzer (Agilent E8364B). In coherent control experiments, both control and signal antennas (Schwarzbeck BBHA 9120D) were equipped with collimating lenses and were fed with the same signal through identical cables. The minimum angle of incidence of *θ* = 13° resulted from the physical size of control antenna (Schwarzbeck BBHA 9120D) and receiving antenna (Schwarzbeck STLP 9148), which had to be placed next to each other.

### Numerical modeling

Simulations were conducted using a full three-dimensional Maxwell finite element method solver in the frequency domain. In case of the (quasi-)normal incidence experiments for optical anisotropy and intrinsic 3D chirality, the simulations correspond to *θ* = 0° (Figs 3b,d and 4b,d). In case of extrinsic 3D chirality, the angle of incidence in the simulations was *θ* = 30°, just like in the experiments (Figs 5b,d and 6b,d). Modeled and experimental metamaterial resonance frequencies are close, but slightly shifted relative to each other. Therefore we show the simulated phase dependence of the signal output beam intensity and polarization for the spectral feature that corresponds to the experimental data, which is within 4% of the experimental frequency in all cases (Figs 3d, 4d, 5d, 6d).

### Definitions

The phase difference *α* between control and signal beams is defined as increasing with increasing optical length of the control beam path, where in-phase excitation corresponds to the anti-node of electric field in the metamaterial plane. Counterclockwise polarization azimuth rotation as seen looking into the beam is defined as positive. A polarization state is defined as right-handed with positive ellipticity angle when the electric field vector at a fixed point in space rotates clockwise as seen by an observer looking into the beam. The input intensities of the signal and control beams are considered 100%, each, so that the total output intensity is 200% in absence of absorption.

## Author Contributions

N.I.Z. and E.P. conceived the idea of the experiment, wrote the paper and supervised the work; E.P. designed the experiment; S.A.M carried out the measurements; J.S. performed numerical modeling; all authors discussed the results and analyzed the data.

## Supplementary Material

Supplementary Information Theoretical description of coherent control of polarization effects in metamaterials

## Figures and Tables

**Table 1:** Interference of coherent beams at oblique incidence

	*E* anti-node (*α* = 0°)	*E* node (*α* = 180°)
p-polarization	*E*_||_ = 2*E*_0_ cos(*θ*)	*E*_||_ = 0
	*B*_||_ = 0	*B*_||_ = 2*B*_0_
	*E*_⊥_ = 0	*E*_⊥_ = 2*E*_0_ sin(*θ*)
	*B*_⊥_ = 0	*B*_⊥_ = 0
s-polarization	*E*_||_ = 2*E*_0_	*E*_||_ = 0
	*B*_||_ = 0	*B*_||_ = 2*B*_0_ cos(*θ*)
	*E*_⊥_ = 0	*E*_⊥_ = 0
	*B*_⊥_ = 2*B*_0_ sin(*θ*)	*B*_⊥_ = 0

## References

[b1] Fedotov V. A., Prosvirnin S. L., Rogacheva A. V. & Zheludev N. I. Mirror that does not change the phase of reflected wave. Appl. Phys. Lett. 88, 091119 (2006).

[b2] Munk B. A. Frequency Selective Surfaces: Theory and Design. Wiley-Interscience, 1st edition, (2000).

[b3] Zheludev N. I., Plum E. & Fedotov V. A. Metamaterial polarization spectral filter: isolated transmission line at any prescribed wavelength. Appl. Phys. Lett. 99, 171915 (2011).

[b4] Strikwerda A. C. *et al.* Comparison of birefringent electric split-ring resonator and meanderline. Opt. Express 17, 136–149 (2009). 19129881 10.1364/oe.17.000136

[b5] Ahn S. W. *et al.* Fabrication of a 50 nm half-pitch wire grid polarizer using nanoimprint lithography. Nanotechnology 16, 1874 (2005).

[b6] Noginov M., Lapine M., Podolskiy V. & Kivshar Y. Focus issue: hyperbolic metamaterials. Opt. Express 21, 14895 (2013). 23787677 10.1364/OE.21.014895

[b7] Rogacheva A. V., Fedotov V. A., Schwanecke A. S. & Zheludev N. I. Giant gyrotropy due to electromagnetic-field coupling in a bilayered chiral structure. Phys. Rev. Lett. 97, 177401 (2006). 17155505 10.1103/PhysRevLett.97.177401

[b8] Plum E., Fedotov V. A., Schwanecke A. S., Zheludev N. I. & Chen Y. Giant optical gyrotropy due to electromagnetic coupling. Appl. Phys. Lett. 90, 223113 (2007).

[b9] Kuwata-Gonokami M. *et al.* Giant optical activity in quasi-two-dimensional planar nanostructures. Phys. Rev. Lett. 95, 227401 (2005). 16384264 10.1103/PhysRevLett.95.227401

[b10] Decker M., Klein M. W., Wegener M. & Linden S. Circular dichroism of planar chiral magnetic metamaterials. Opt. Lett. 32, 856–858 (2007). 17339960 10.1364/ol.32.000856

[b11] Plum E., Fedotov V. A. & Zheludev N. I. Optical activity in extrinsically chiral metamaterial. Appl. Phys. Lett. 93, 191911 (2008).

[b12] Plum E. *et al.* Metamaterials: optical activity without chirality. Phys. Rev. Lett. 102, 113902 (2009). 19392202 10.1103/PhysRevLett.102.113902

[b13] Yu N. *et al.* Light propagation with phase discontinuities: generalized laws of reflection and refraction. Science 334, 333–337 (2011). 21885733 10.1126/science.1210713

[b14] Rogers E. T. F. *et al.* A super-oscillatory lens optical microscope for subwavelength imaging. Nature Mater. 11, 432–435 (2012). 22447113 10.1038/nmat3280

[b15] Ni X., Ishii S., Kildishev A. V. & Shalaev V. M. Ultra-thin, planar, Babinet-inverted plasmonic metalenses. Light: Science and Applications 2, e72 (2013).

[b16] Stockman M. I., Faleev S. V. & Bergman D. J. Coherent control of femtosecond energy localization in nanosystems. Phys. Rev. Lett. 88, 067402 (2002). 11863849 10.1103/PhysRevLett.88.067402

[b17] Li X. & Stockman M. I. Highly efficient spatiotemporal coherent control in nanoplasmonics on a nanometer-femtosecond scale by time reversal. Phys. Rev. B 77, 195109 (2008).

[b18] Choi S. B. *et al.* Directional control of surface plasmon polariton waves propagating through an asymmetric Bragg resonator. Appl. Phys. Lett. 94, 063115 (2009).

[b19] Utikal T., Stockman M. I., Heberle A. P., Lippitz M. & Giessen H. All-optical control of the ultrafast dynamics of a hybrid plasmonic system. Phys. Rev. Lett. 104, 113903 (2010). 20366478 10.1103/PhysRevLett.104.113903

[b20] Kao T. S., Jenkins S. D., Ruostekoski J. & Zheludev N. I. Coherent control of nanoscale light localization in metamaterial: creating and positioning a sub-wavelength energy hot-spot. Phys. Rev. Lett. 106, 085501 (2011). 21405581 10.1103/PhysRevLett.106.085501

[b21] Gjonaj B. *et al.* Active spatial control of plasmonic fields. Nature Photon. 5, 360–363 (2011).

[b22] Wan W. *et al.* Time-reversed lasing and interferometric control of absorption. Science 331, 889 (2011). 21330539 10.1126/science.1200735

[b23] Li Z., Zhang S., Halas N. J., Nordlander P. & Xu H. Coherent modulation of propagating plasmons in silver-nanowire-based structures. Small 7, 593–596 (2011). 21370461 10.1002/smll.201001775

[b24] Miyata M. & Takahara J. Excitation control of long-range surface plasmons by two incident beams. Opt. Express 20, 9493–9500 (2012). 22535040 10.1364/OE.20.009493

[b25] Yoon J. W., Koh G. M., Song S. H. & Magnusson R. Measurement and modeling of a complete optical absorption and scattering by coherent surface plasmon-polariton excitation using a silver thin-film grating. Phys. Rev. Lett. 109, 257402 (2012). 23368498 10.1103/PhysRevLett.109.257402

[b26] Brinks D., Castro-Lopez M., Hildner R. & van Hulst N. F. Plasmonic antennas as design elements for coherent ultrafast nanophotonics. PNAS 110, 18386 (2013). 24163355 10.1073/pnas.1308652110 PMC3832020

[b27] Zhang J., MacDonald K. F. & Zheludev N. I. Controlling light-with-light without nonlinearity. Light: Science and Applications 1, e18 (2012).

[b28] Shi J. *et al.* Coherent control of Snell's law at metasurfaces. Opt. Express 22, 21051 (2014). 25321305 10.1364/OE.22.021051

[b29] Roger T. *et al.* Coherent modulation of single photons using graphene and plasmonic metamaterials. In: 5th International Conference on Metamaterials, Photonic Crystals and Plasmonics (META'14), Singapore, (2014).

[b30] Mousavi S. A., Plum E., Shi J. & Zheludev N. I. Coherent control of birefringence and optical activity. Appl. Phys. Lett. 105, 011906 (2014).

[b31] Fang X., Tseng M. L., Tsai D. P. & Zheludev N. I. Coherent excitation-selective spectroscopy in planar metamaterials. arXiv, 1312.0524 (2013).

[b32] Kelvin L. Baltimore Lectures on Molecular Dynamics and the Wave Theory of Light, 619. C.J. Clay and Sons, Cambridge University Press Warehouse, London (1904).

[b33] Pasteur L. Mémoire sur la relation qui peut exister entre la forme cristalline et la composition chimique, et sur la cause de la polarization rotatoire. C. R. Acad. Sci. Paris 26, 535–539 (1848).

[b34] Bose J. C. On the rotation of plane of polarization of electric waves by a twisted structure. Proceedings of the Royal Society of London 63, 146–152 (1898).

[b35] Lindman K. F. Über eine durch ein isotropes System von spiralförmigen Resonatoren erzeugte Rotationspolarisation der elektromagnetischen Wellen. Ann. Phys. 368, 621–644 (1920).

[b36] Gansel J. K. *et al.* Gold helix photonic metamaterial as broadband circular polarizer. Science 325, 1513–1515 (2009). 19696310 10.1126/science.1177031

[b37] Svirko Y., Zheludev N. & Osipov M. Layered chiral metallic microstructures with inductive coupling. Appl. Phys. Lett. 78, 498–500 (2001).

[b38] Plum E. *et al.* Metamaterial with negative index due to chirality. Phys. Rev. B 79, 035407 (2009).

[b39] Zhou J. *et al.* Negative refractive index due to chirality. Phys. Rev. B 79, 121104(R) (2009).

[b40] Decker M. *et al.* Strong optical activity from twisted-cross photonic metamaterials. Opt. Lett. 34, 2501–2503 (2009). 19684829 10.1364/ol.34.002501

[b41] Bunn C. W. Chemical Crystallography, 88. Oxford University Press, New York (1945).

[b42] Williams R. Optical rotatory effect in the nematic liquid phase of p-azoxyanisole. Phys. Rev. Lett. 21, 342–344 (1968).

[b43] Fedotov V. A. *et al.* Asymmetric propagation of electromagnetic waves through a planar chiral structure. Phys. Rev. Lett. 97, 167401 (2006). 17155432 10.1103/PhysRevLett.97.167401

[b44] Singh R., Plum E., Zhang W. & Zheludev N. I. Highly tunable optical activity in planar achiral terahertz metamaterials. Opt. Express 18, 13425–13430 (2010). 20588473 10.1364/OE.18.013425

[b45] Ren M., Plum E., Xu J. & Zheludev N. I. Giant nonlinear optical activity in a plasmonic metamaterial. Nature Comms. 3, 833 (2012). 10.1038/ncomms1805 22588295

