# Supplementary material for: Coherent control of optical polarization effects in metamaterials
Source: Sci Rep. 2015 Mar 10;5:8977. doi: 10.1038/srep08977 (PMC4354045; doi:10.1038/srep08977)
Supplement: Supplementary Information — Theoretical description of coherent control of polarization effects in metamaterials [file srep08977-s1.pdf]

Supplementary information: Coherent control of optical polarization effects in metamaterials

Seyedmohammad A. Mousavi,[1] Eric Plum,[1,*] Jinhui Shi,[1,2] and Nikolay I. Zheludev[1,3,†]

[1] *Optoelectronics Research Centre and Centre for Photonic Metamaterials, University of Southampton, SO17 1BJ, UK*
[2] *Key Laboratory of In-Fiber Integrated Optics of Ministry of Education, College of Science, Harbin Engineering University, Harbin 150001, China*
[3] *Centre for Disruptive Photonic Technologies, Nanyang Technological University, Singapore 637378, Singapore*

The coherent interaction of electromagnetic waves on thin functional linear materials can be described by Jones transmission and scattering matrices t and s. Here we consider signal $E^{\rm in}$ and control $E^{\rm in}e^{i\alpha}$ input beams with a phase difference α that are incident on opposite sides of a thin linear material as described in the main manuscript. The signal output $E^{\rm s,out}$ is formed by the superposition of the transmitted signal beam $tE^{\rm in}$ and the scattered (reflected) control beam $sE^{\rm in}e^{i\alpha}$. Similarly, the control output $E^{\rm c,out}$ is the superposition of the scattered signal and transmitted control input beams. Therefore,

$$\begin{aligned} E^{\rm s,out} &= \overrightarrow{t} E^{\rm in} + \overleftarrow{s} E^{\rm in} e^{i\alpha}, \\ E^{\rm c,out} &= \overrightarrow{s} E^{\rm in} + \overleftarrow{t} E^{\rm in} e^{i\alpha}, \end{aligned} \tag{1}$$

where right and left arrows mark Jones matrices for the directions of the incident signal an control beams, respectively. In case of planar metamaterials, transmission simply corresponds to the superposition of incident and scattered fields, i.e. $t = 1 + s$, with 1 being the unity matrix.

A. Coherent control of manifestations of optical anisotropy

Optical anisotropy has been studied for a planar metamaterial ($t = 1 + s$). Planar anisotropic metamaterials are described by the same ordinary and extraordinary scattering coefficients s_x and s_y for both directions of illumination and therefore equation (1) simplifies to

$$\begin{aligned} E_j^{\rm s,out} &= \left[(1+s_j) + s_j e^{i\alpha_j}\right] E_j^{\rm in}, \\ E_j^{\rm c,out} &= \left[s_j + (1+s_j)e^{i\alpha_j}\right] E_j^{\rm in}. \end{aligned} \tag{2}$$

where $j = x, y$. From here, it is clear that the corresponding input and output beams must be identical for $\alpha = 180°$, when $e^{i\alpha} = -1$, implying complete transparency at the magnetic node, as observed in our experiments and simulations. It also follows that the output intensities will have a sinusoidal phase dependence, which can be written in the general form $I^{\rm out} = A\cos^2\left(\frac{\alpha-\alpha_0}{2}\right) + C$ and has been seen experimentally and numerically.

B. Coherent control of manifestations of optical activity

Optical activity is most easily characterized in terms of its circular polarization eigenstates. Simple constructive or destructive interference occurs for incident control and signal polarizations that have the same projection onto the metamaterial plane. As the handedness of polarization states is measured looking into the beam, this implies that coherent control is achieved with signal and control beams of opposite handedness. Thus equation (1) becomes

$$\begin{aligned} E_\pm^{\rm s,out} &= \overrightarrow{t}_\pm E_\pm^{\rm s,in} + \overleftarrow{s}_\mp E_\mp^{\rm c,in}, \\ E_\mp^{\rm c,out} &= \overrightarrow{s}_\pm E_\pm^{\rm s,in} + \overleftarrow{t}_\mp E_\mp^{\rm c,in}, \end{aligned} \tag{3}$$

with $E_\mp^{\rm c,in} = E_\pm^{\rm s,in} e^{i\alpha_\pm}$, where indices "+" and "-" stand for right-handed and left-handed circular polarizations.

---

*Electronic address: erp@orc.soton.ac.uk
†Electronic address: niz@orc.soton.ac.uk; URL: www.nanophotonics.org.uk

The polarization states of the input and output beams are defined by their azimuth Φ and ellipticity angle η

$$\Phi = -\frac{1}{2}[\arg(E_+) - \arg(E_-)], \tag{4}$$

$$\eta = \frac{1}{2}\arcsin\left(\frac{|E_+|^2 - |E_-|^2}{|E_+|^2 + |E_-|^2}\right). \tag{5}$$

C. Intrinsic 3D chirality

In the simplest case, an intrinsically 3D-chiral metamaterial is characterized by a polarization-independent scattering coefficient s_0 and transmission coefficients t_+ and t_-, which are identical for opposite directions of illumination. Simulations show that this holds for the pair of twisted crosses discussed in the main manuscript, while it becomes an approximation at resonances in our experimental case, where resonant reflectivity is slightly different for opposite sides of the sample. Based on the above, equation (3) simplifies to

$$\begin{aligned} E_\pm^{\mathrm{s,out}} &= \left(t_\pm + s_0 e^{i\alpha_\pm}\right) E_\pm^{\mathrm{s,in}}, \\ E_\pm^{\mathrm{c,out}} &= \left(t_\pm e^{i\alpha_\mp} + s_0\right) E_\mp^{\mathrm{s,in}}. \end{aligned} \tag{6}$$

For linear polarization, we can always choose coordinates so that $E_+^{\mathrm{s,in}} = E_-^{\mathrm{s,in}}$ and $\alpha_+ = \alpha_-$. In this case it is apparent from equations (4) and (5) that the control output at phase α is equivalent to the signal output at phase $-\alpha$ with the same azimuth rotation and ellipticity. The same conclusion has been drawn from symmetry arguments in the main manuscript.

D. Extrinsic 3D chirality

Extrinsic 3D chirality has been studied for planar metamaterials ($t = 1 + s$). In the limit of a vanishing anisotropic response the extrinsically 3D-chiral metamaterials have circular polarization eigenstates and are characterized by scattering coefficients s_+ and s_- for the incident signal beam. As extrinsic chirality is reversed for the direction of the control beam, $\overleftarrow{s_+} = s_-$ and $\overleftarrow{s_-} = s_+$, so that equation (3) simplifies to

$$\begin{aligned} E_\pm^{\mathrm{s,out}} &= \left[(1 + s_\pm) + s_\pm e^{i\alpha_\pm}\right] E_\pm^{\mathrm{s,in}}, \\ E_\mp^{\mathrm{c,out}} &= \left[(1 + s_\pm) e^{i\alpha_\pm} + s_\pm\right] E_\pm^{\mathrm{s,in}}. \end{aligned} \tag{7}$$

It follows from equations (4) and (5) that the control output at phase α corresponds to the signal output at phase $-\alpha$ with reversed azimuth rotation and ellipticity. The same conclusion has been drawn in the main manuscript based on symmetry arguments for planar metamaterials in general. Considering $E_\mp^{\mathrm{c,in}} = E_\pm^{\mathrm{s,in}} e^{i\alpha_\pm}$, the corresponding input and output beams must be identical for $\alpha_\pm = 180°$, when $e^{i\alpha_\pm} = -1$, implying complete transparency at the magnetic node as observed in our experiments and simulations. It also follows that the output intensities will have a sinusoidal phase dependence, which can be written in the general form $I^{\mathrm{out}} = A\cos^2\left(\frac{\alpha-\alpha_0}{2}\right) + C$ and has been observed experimentally and numerically.
